# Why many studies of individual differences with inhibition tasks may not localize correlations

**DOI:** 10.3758/s13423-023-02293-3

**Published:** 2023-07-05

**Authors:** Jeffrey N. Rouder, Aakriti Kumar, Julia M. Haaf

**Affiliations:** 1 University of California, Irvine, CA USA; 2 University of Amsterdam, Amsterdam, Netherlands

**Keywords:** Individual differences, Cognitive tasks, Hierarchical models, Bayesian inference

## Abstract

Individual difference exploration of cognitive domains is predicated on being able to ascertain how well performance on tasks covary. Yet, establishing correlations among common inhibition tasks such as Stroop or flanker tasks has proven quite difficult. It remains unclear whether this difficulty occurs because there truly is a lack of correlation or whether analytic techniques to localize correlations perform poorly real-world contexts because of excessive measurement error from trial noise. In this paper, we explore how well correlations may localized in large data sets with many people, tasks, and replicate trials. Using hierarchical models to separate trial noise from true individual variability, we show that trial noise in 24 extant tasks is about 8 times greater than individual variability. This degree of trial noise results in massive attenuation in correlations and instability in Spearman corrections. We then develop hierarchical models that account for variation across trials, variation across individuals, and covariation across individuals and tasks. These hierarchical models also perform poorly in localizing correlations. The advantage of these models is not in estimation efficiency, but in providing a sense of uncertainty so that researchers are less likely to misinterpret variability in their data. We discuss possible improvements to study designs to help localize correlations.

In 1957, Lee Cronbach gave a presidential address to the American Psychological Association where he advocates merging two major but separate traditions in research psychology (Cronbach, 1957). One was termed the *correlational tradition*, and it referred to the rapid advances in psychometrics and scaling at the time. The other was the *experimental tradition,* which is readily recognizable in this journal and several others. Although these traditions remain largely separate today, one area where there has been substantial merging is the study of individual differences in cognitive control. Individual-difference studies often include true experimental tasks such as the Stroop task (Stroop, 1935) Simon task (Simon, 1968) the Flanker task (Eriksen and Eriksen, 1974) face of it, individual-difference researchers should be sanguine about using such tasks for the following reasons: First, many of these tasks are designed to isolate a specific cognitive process, such as cognitive control, and they do so by contrasting specific conditions. For example, in the Stroop task, the score is the contrast between performance for incongruent and congruent conditions. The subtraction inherent in the contrast controls for unrelated sources of variation such as overall speed. Second, many of these tasks are robust in that the effects are easy to obtain in a variety of circumstances. Take again, for example, the Stroop task. The Stroop effect is so robust that it is considered universal (MacLeod, 1991). Third, because these tasks are laboratory based and center on experimenter-controlled manipulations, they often have a high degree of internal validity. Fourth, because these tasks are used so often, there is usually a large literature about them to guide implementation and interpretation. Fifth, task scores are relatively easy to collect and analyze with latent-variable models.

Before going on, we ask the reader to draw a sharp distinction between a *task* and a *measure.* Tasks are true experiments where Donders’ subtractive logic (Donders, 1868) is used to localize a process of interest. Two conditions are constructed where the only difference between them that is that the process of interest loads more highly on to one than the other. The contrast of the conditions allows for a measure of the process free from nuisance factors. Measures are instruments that do not have conditions nor use Donders’ subtraction method. Good examples of measures are the the anti-saccade accuracy measure (Kane et al., 2001), the N-back memory measure (Cohen et al., 1994), and the stop-signal measure (Logan and Cowan, 1984; Verbruggen et al., 2019). Measures typically reflect the composite of several skills and processes including but not limited to cognitive control. For example, obtaining high accuracy in the antisaccade measure requires not only suppression of the prepotent orienting response to the cue, but also speed in moving ones eyes and speed in target identification. In our usage, measures are not true experiments. They do not have an associated experimental manipulation and a contrast. Moreover, the claim that they index a particular process is made *prima facie* and without recourse to experimental logic. This does not mean that the claim is undesirable. It does mean that it is up to researchers to assess the claim by their own standard for appropriateness without recourse to an underlying experimental logic.

**Fig. 1:**
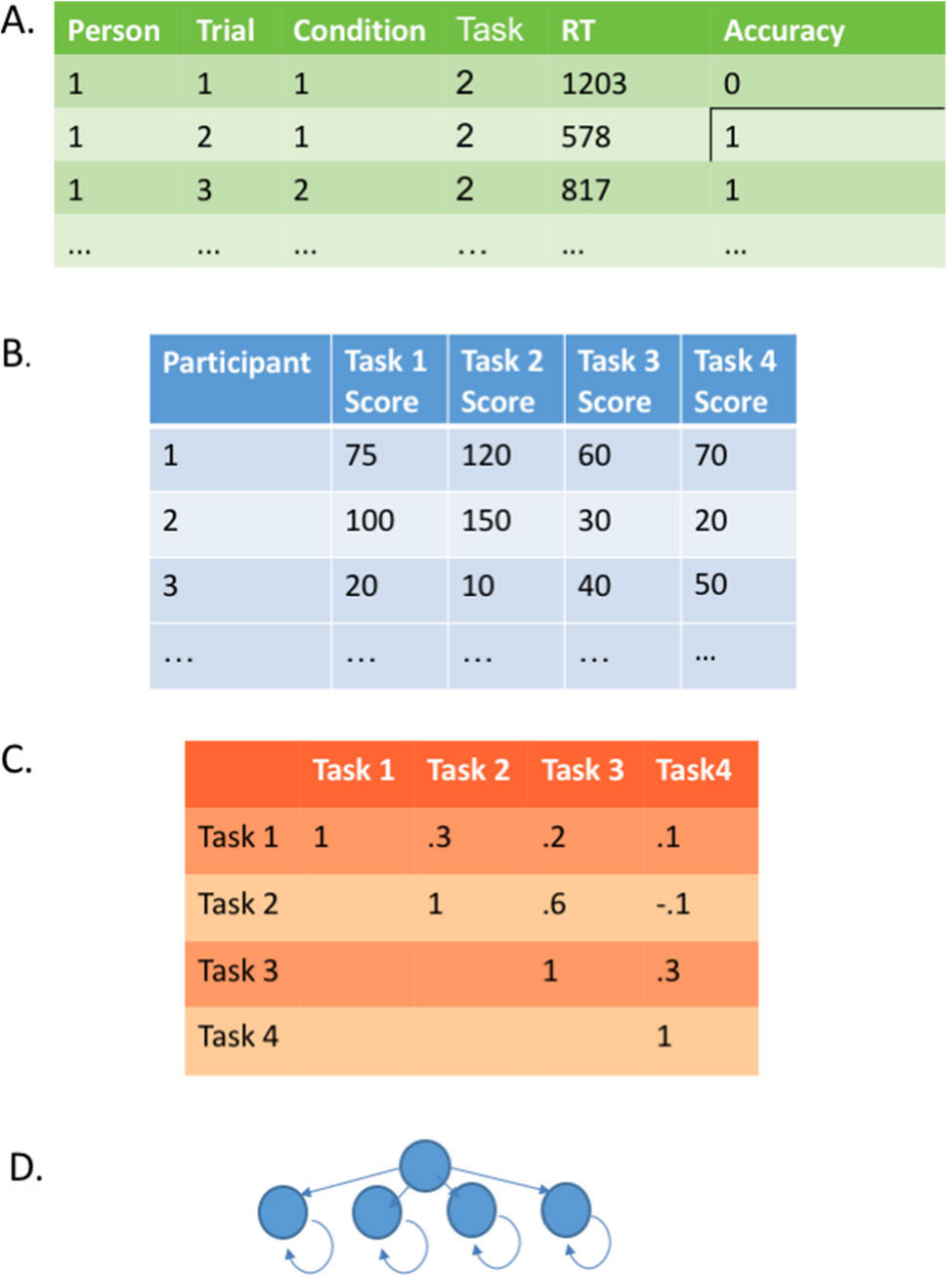
In the usual course of analysis, the raw data (A) are used to tabulate sample effects (B). The covariation among these task-by-person sample effects (C) then serve as input to latent variable modeling (D)

Figure 1 shows the usual course of analysis in individual-difference research with cognitive tasks. There are raw data (Panel A), which are quite numerous, often on the order of hundreds of thousands of observations. These are cleaned, and to start the analysis, task scores for each participant are tabulated (Panel B). For example, if Task 1 is a Stroop task, then the task scores would be each individual’s Stroop effect, that is, the difference between the mean RT for incongruent and congruent conditions. A typical task score is a difference of conditions, and might be in the 10s of milliseconds range. The table of individual task scores is treated as a multivariate distribution, and the covariation of this distribution (Panel C) is decomposed into meaningful sources of variation through latent variable models (Panel D; e.g., Bollen 1989; Skrondal and Rabe-Hesketh 2004).

**Table 1:** The correlation between a Stroop and Flanker tasks for selected publications

Study	Year	Correlation	Source
Friedman & Miyake	2004	0.18	Cited in Text
Unsworth & Spillers	2010	0.17	Cited in Text
Unsworth & McMillan	2014	0.22	Cited in Text
Shipstead et al.	2014	0.11	Cited in Text
Pettigrew & Martin	2014	0.03	Cited in Text
Shipstead et al.	2015	0.23	Cited in Text
Redick et al.	2015	0.17	Cited in Text
Von Bastian et al.	2016	0	Computed
Hedge et al.	2018	-0.05	Recomputed
Rey-Mermet et al. (ave)	2018	0	Recomputed
Whitehead et al. (ave)	2019	0.03	Recomputed
Draheim et al.	2020	0.17	Cited in Text

*Note.* Recomputed correlations may differ from original source due to differences in cleaning steps

The above latent-variable approach to individual differences has been successful in some domains, such as personality, where rich factor structures are used to capture individual differences (Ashton et al., 2004; McCrae and Costa Jr, 1997). Indeed, it seemed some twenty years ago that the same strategy would succeed in cognitive control (Kane and Engle, 2003; Miyake et al., 2000). Yet, the latent-variable approach has not lived up to the promise, at least not in our opinion. Scores from experimental tasks correlate with one another far less than one might think *a priori.* Take the correlation between Stroop and flanker tasks, two popular tasks for measuring inhibition. Table 1 shows some published values from the literature. As a rule, effects in inhibition tasks (as opposed to measures) show low correlations (Rey-Mermet et al., 2018). Indeed, even if we cherry pick the high end of these correlations, we tend not to find values about .3.

**Fig. 2:**
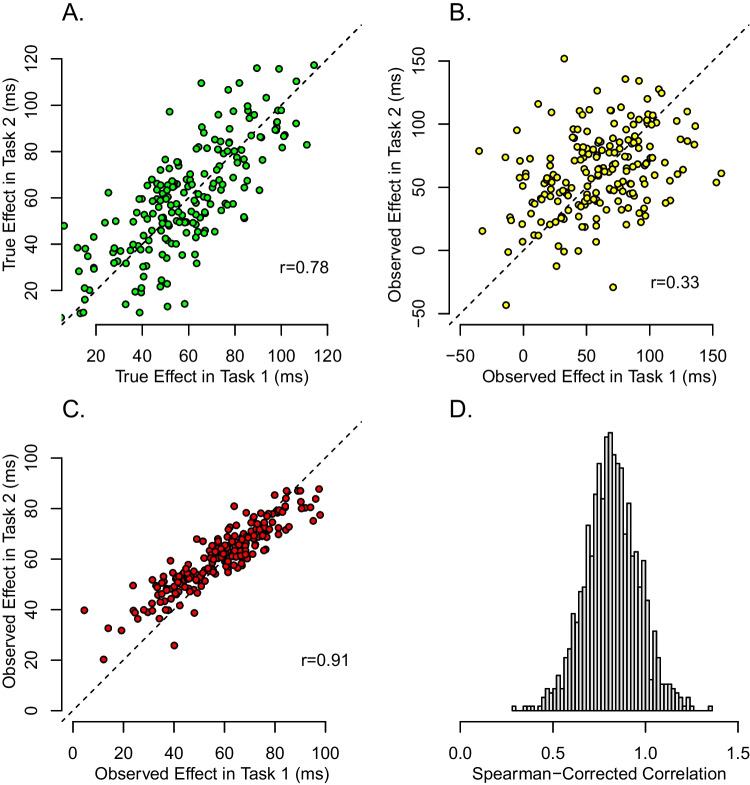
The effects of trial variability on the assessment of correlations among tasks. A: Hypothetical true individual effects show a large degree of correlation across two tasks. B: Observed effects are so perturbed by trial variability that the correlation is greatly attenuated. C: Hierarchical model recovery for the data in A. D: Spearman correction-for-attenuation in a small simulation with realistic settings

The question of why these correlations are low has been the subject of recent work by Draheim et al. (2019); Enkavi et al. (2019); Hedge et al. (2018) and Rey-Mermet et al. (2018) among others. On one hand, they could reflect underlying true task performance that is uncorrelated or weakly correlated. In this case, the low correlations indicate that performance on the tasks do not largely overlap, and that the tasks are indexing different mental processes. Indeed, this substantive interpretation is taken by Rey-Mermet et al. (2018), who argue that inhibition should be viewed as a disparate rather than a unified concept. By extension, different tasks rely on different and disparate inhibition processes.

On the other hand, the true correlations could be large but masked by measurement error. Several authors have noted the possibility of a large degree of measurement error. Hedge et al. (2018), for example, set out to empirically assess the reliability of task measures by asking participants to perform a battery of tasks and to return three weeks later to repeat the battery. With these two measures, Hedge et al. (2018) computed the test-retest reliability of the tasks. The results were somewhat disheartening with test-retest reliabilities for popular tasks in the range from .2 to .7. Draheim et al. (2019) argue that commonly used response time difference scores are susceptible to low reliability and other artifacts such as speed-accuracy tradeoffs.

It has been well known for over a century that correlations among measures are attenuated in low reliability environments (Spearman, 1904). Yet, how much attenuation can we expect? If it is negligible, then the observed low correlations may be interpreted as true indicators that the tasks are largely measuring uncorrelated mental abilities. But if the attenuation is sizable, then the underlying true correlation remains unknown. One of our contributions in this paper is to document just how big this attenuation is in common designs.

Figure 2 provides an example of attenuation. Shown in Panel A are hypothetical *true difference scores* (or true effects) for 200 individuals on two tasks. The plot is a scatter plot—each point is for an individual; the x-axis value is the true score on one task, the y-axis value is the true score on the other task. As can be seen, there is a large correlation, in this case it is 0.78. Researchers do not observe these true scores; instead they analyze difference scores from noisy trial data with the tabulation shown in Fig. 1. Figure 2B shows the scatterplot of these observed difference scores (or observed effects). Because these observed effects reflect trial noise, the correlation is attenuated. In this case it is 0.33. While this correlation is statistically detectable, the value is dramatically lower than the true one.

The amount of attenuation of the correlation is dependent on critical inputs such as the number of trials and the degree of trial variability. Therefore, to get a realistic picture of the effects of measurement error it is critical to obtain realistic values for these inputs. In this paper, we survey 15 fairly large inhibition studies. From this survey, presented here subsequently, we derive typical values for the number of trials and the degree of trial variability. These typical values are used in Fig. 2, and the amount of attenuation of the correlation therefore represents a typical rather than a worst-case scenario. As will be discussed, we believe that observed correlations in typical designs are less than 1/2 of the true values.

## The role of trial noise

The amount of attenuation shown in Fig. 2, from 0.78 to 0.33, is striking. No wonder it has been so hard to find correlations! What can be done?

One of the key features of inhibition tasks is that they are comprised on trials. While the responses on any given trial may be noisy, this noise may be overcome by running many trials. If there are a great many trials, then the sample means precisely estimate true means, sample differences precisely estimate true differences, and the correlation reflects the true correlation among the tasks. If there are few trials, then the sample means are variable and the observed correlation is attenuated. Hence, the number of trials per task is a critical quantity as it determines the systematic downward bias in correlation.

There are two immediate consequences to having multiple trials (Rouder and Haaf, 2019). The first is that one cannot talk about the reliability of a task or the correlation among two tasks. These values are critically dependent on the number of trials (called hereforth *trial size*). We cannot compare different values from different experiments without somehow accounting for differences in this design element. Simply put, there is no such thing as the reliability of a task or a correlation between tasks without reference to trial size. The second consequence is that trial size far more important than the number of participants in interpreting results. The number of participants determines the unsystematic noise in the correlation; the trial size determines the systematic downward bias. With few trials per task and many participants, researchers will have high confidence in a greatly biased estimate.

There are two potential benefits to understanding the role of trial noise and trial size. The first is that trial noise can always be overcome in designs with large trials sizes. We discuss the pragmatics of this approach in the general discussion. The second benefit is that by using hierarchical models, trial noise may be modeled and removed. For example, Behseta, Berdyyeva, Olson, and Kass (2009), Haines et al. (2020); Matzke et al. (2017); Rouder and Haaf (2019) and Whitehead, Brewer, and Blais (2020) each propose hierarchical models to disattenuate correlations with limited trial sizes. The potential of such models is shown in Fig. 2C. Here, a hierarchical model, to be discussed subsequently, was applied to the data in 2B, and the resulting posterior estimates of participants’ effects reveal the true strong correlation. We refer to *localization* of correlation. When estimates of correlation are precise and accurate, we say correlations are *well localized*. When estimates are either highly attenuated or imprecise, then estimates are poorly *localized.*. A suitable measure of localizaiton is root-mean-square error (RMSE) between an estimate and a true value because this measure captures both bias and imprecision.

Based on the demonstration in Fig. 2C, we had come into this research with the hope of telling a *you-can-have-your-cake-and-eat-it* story. We thought that perhaps hierarchical models would allow for the accurate localization of correlations in typical designs providing for an answer to whether cognitive control is unified or disparate. Yet, the story we tell here is far more complicated. To foreshadow, overall estimates from hierarchical models do disattenuate correlations. But, in the course, they suffer from a large degree of imprecision. It seems that in typical designs, one can use sample statistics and suffer massive attenuation or use a modeling approach and accept a large degree of imprecision. And this difficulty is why we believe most studies of individual differences with inhibition tasks fail to localize correlations. This story is not the one we had hoped to tell. It would have been so much more desirable if we could show that the models we have develped and advocated for solve such critical problems. But we cannot do so. The inability to localize correlations even with the most sophisticated statistical approaches is an important story for the community of individual-differences scholars.

## Spearman’s correction for attenuation

Before addressing the main question about localizing correlations, we consider the Spearman (1904) correction for the attenuation. In this brief detour, we assess whether Spearman’s correction leads to the localization of latent correlations among tasks in typical designs. The assessment provides guidance because the data generation in simulations match well with the assumptions in Spearman’s correction. If Spearman’s correction cannot localize the latent correlations in realistic designs, these correlations may indeed be unrecoverable.

Spearman’s derivation comes from decomposing observed variation into true variation and measurement noise. When reliabilities are low, correlations may be upweighted to account for them. In Spearman’s classic formula, the disattenuated correlation, denoted $r'_{xy}$ between two variables *x* and *y* is

$$\begin{aligned} r'_{xy} = \frac{r_{xy}}{\sqrt{r_{xx}r_{yy}}}, \end{aligned}$$

where $r_{xy}$ is the sample correlation and $r_{xx}$ and $r_{yy}$ are the sample reliabilities.^1^

Spearman’s correction, while well known, is not used often. The problem is that it is unstable. Panel D of Fig. 2 shows the results of a small simulation based on realistic values from inhibition tasks discussed subsequently. The true correlation is .80. The Spearman-corrected correlations, however, are not only variable ranging from 0.38 to 1.72, but not restricted to valid ranges. In fact, 10.10% of the simulated values are greater than 1.0. We should take these problems with Spearman’s correction seriously. The poor results in Fig. 2D may indicate that in low-reliability environments, true correlations among tasks may not be localized. And this lack of localization may be fundamental—trial noise may destroy the correlation signatures in designs with limited trial sizes.

In this paper, we explore how well correlations may be localized with the conventional analysis (Fig. 1), with the Spearman correction, and with hierarhical models. We make our main claims by simulating data from known true correlation values. We then see how well the methods estimate these true values. For these simulations to be useful, they must be realistic. The simulated data must not only be from realistic designs, but they must have realistic levels of true individual variation and of true trial noise. The simulations are only as good as these inputs.

To make sure our simulations are useful, we analyze existing data sets to find appropriate settings for simulations. This analysis is presented in the next section. With these settings established, we simulate data and assess whether correlations are recoverable. The hierarchical latent correlation estimators, while far from perfect, are better than Spearman-corrected correlation estimators. Subsequently, we apply the same analysis to a large data set from Rey-Mermet et al. (2018) spanning four inhibition tasks to assess whether the observed low correlations reflect independent task performance or attenuation from trial noise. Yet, even with hierarchical modeling, we are unable to definitively answer this question.

## Variability in experimental tasks

To use simulations to assess how well correlations may be localized, it is important to understand typical ranges of variability. Our approach is to gather a reasonable corpus of studies and analyze them, one-at-time, to understand the levels of true individual variation and trial noise. There are two issues: A. How to measure these levels of variation?, and B. Which extant studies to analyze? We take them in turn:

### One-task measurement model

To estimate within-trial and across-individual variabilities, we use an ordinary variance-components hierarchical model. To appreciate how variation can be assessed, the models need to be fully specified rather than left to short-hand. Let $Y_{ijk\ell }$ be the $\ell$th response for the *i*th individual in the *j*th task and *k*th condition. In this section we analyze each task independently, so we may safely ignore *j*, the task subscript (we will use it subsequently, however). The model for one task is:

$$\begin{aligned} Y_{ik\ell } \sim \text {Normal}(\alpha _i+x_k\theta _i,\sigma ^2), \end{aligned}$$

where $\alpha _i$ is the *i*th individual’s true response time in the congruent condition, $x_k=0,1$ codes for the incongruent condition, $\theta _i$ is the *i*th individual’s true effect, and $\sigma ^2$ is the trial noise within an individual-by-condition cell. The critical parameters are the $\theta _i$s, and these are modeled as random effects:

$$\begin{aligned} \theta _i \sim \text {Normal}(\mu _\theta ,\sigma ^2_\theta ), \end{aligned}$$

where $\mu _\theta$ describes the overall mean effect and $\sigma ^2_\theta$ is the between-person variation in individuals’ true effects. The two variabilities are the within-cell trial noise, $\sigma ^2$, and between-individual variance, $\sigma ^2_\theta$.

**Fig. 3:**
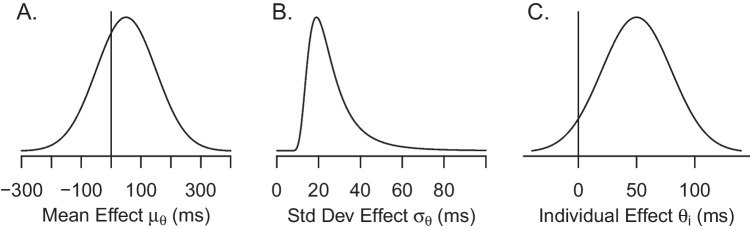
A, B: Prior distributions of $\mu _\theta$ and $\sigma _\theta$, respectively. C: Prior distribution of $\theta _i$ for $\mu _\theta =50$ ms and $\sigma _\theta =30$ ms

To analyze the model priors are needed for all parameters. Our strategy is to choose scientifically-informed priors (Dienes and Mclatchie 2018; Etz, Haaf, Rouder, and Vandekerckhove, 2018; Rouder, Engelhardt, McCabe, and Morey, 2016; Vanpaemel and Lee 2012) that anticipate the overall scale of the data. The parameters on baseline response times, in seconds, are $\alpha _i \sim \text {Normal}(.8,1)$. These priors are quite broad and place no substantive constraints on the data other than baselines are somewhere around 800 ms plus or minus 2000 ms. The prior on variability is $\sigma ^2 \sim \text {Inverse Gamma}(.1,.1)$, where the inverse gamma is parameterized with shape and scale parameters (Rouder and Lu, 2005). This prior, too, is broad and places no substantive constraint on data. Priors for $\mu _\theta$ and $\sigma ^2_\theta$ were informed by the empirical observation that typical inhibition effects are in the range of 10 ms to 100 ms. They were $\mu _\theta \sim \text {Normal}(50, 100^2 )$ and $\sigma ^2_\theta \sim \text {Inverse Gamma}(2,30^2)$, where the values are in milliseconds rather than seconds. A graph of these prior settings for $\mu$ and $\sigma _\theta =\sqrt{\sigma ^2_\theta }$ is shown in Fig. 3. These priors make the substantive assumption that effects are relatively small and are not arbitrarily variable across people. The scale setting on $\sigma ^2_\theta$ is important as it controls the amount of regularization in the model, and the choice of 30 (on the ms scale) is scientifically informed from previous analyses (see Haaf and Rouder 2017).

### Data sets

We applied this model to a collection of 24 experimental tasks from a variety of authors. Brief descriptions of the tasks are provided in the Appendix. It is reasonable to ask why these 24 and whether they are representative. The experiments were chosen based on the following three criteria: I. Raw trial-level data were available and adequately documented. This criterion is necessary because model analysis relies on the raw data and cannot be performed with the usual summary statistics. II. These raw data could be shared. This research is offered within a fully open and transparent mode (Rouder, Haaf, and Snyder, 2019), and you may inspect all steps from raw data to conclusions. III. The data come from an experimental setup where there was a contrast between conditions; i.e., between congruent and incongruent conditions.

The results from applying the one-task measurement model to the 24 sets are shown in Table 2. The first three columns describe the sample sizes: The first column is the total number of observations across the two conditions after cleaning (see Appendix), the second column is the total number of individuals, and the third column is the average number of replicates per individual per condition.

**Table 2:** Sample sizes, reliabilities, variabilities, and the signal-to-noise ratio for 24 cognitive control tasks

	Sample sizes			Reliability		Sample		Parameters		Ratio
	Obs	Indv	Rep	Full	Split	Effect	$s_d$	$\hat{\sigma }$	$\hat{\sigma }_{\theta }$	$\hat{\eta }$
Stroop										
1. von Bastian	11,245	121	46	0.24	0.34	64	47	198	22	0.11
2. Hedge	43,408	53	410	0.83	0.75	69	32	188	29	0.16
3. Pratte i	11,114	38	146	0.61	0.68	91	50	264	36	0.14
4. Pratte ii	12,565	38	165	0.19	-0.07	12	20	160	15	0.10
5. Rey-Mermet i	48,937	264	93	0.40	0.57	54	30	155	18	0.12
6. Rey-Mermet ii	48,966	261	94	0.86	0.84	59	69	174	64	0.36
7. Whitehead i	122,547	178	344	0.70	0.71	76	53	378	44	0.12
8. Whitehead ii	133,966	194	345	0.56	0.63	78	52	408	37	0.09
9. Whitehead iii	125,979	210	300	0.57	0.51	116	50	402	37	0.09
Simon										
10. von Bastian	23,453	121	97	0.60	0.61	79	36	128	28	0.22
11. Pratte i	17,343	38	228	0.46	0.62	17	24	186	18	0.10
12. Pratte ii	12,266	38	161	0.57	0.51	30	30	175	23	0.13
13. Whitehead i	127,036	178	357	0.71	0.72	67	29	205	24	0.12
14. Whitehead ii	139,332	194	359	0.56	0.59	66	26	212	19	0.09
15. Whitehead iii	125,953	210	300	0.66	0.66	102	28	201	23	0.11
Flanker										
16. von Bastian	11,215	121	46	-0.02	-0.55	2	32	152	15	0.10
17. Hedge	43,384	53	409	0.80	0.79	44	16	100	15	0.15
18. Rey-Mermet i	49,300	265	93	0.18	0.17	30	24	147	13	0.09
19. Rey-Mermet ii	39,275	207	95	0.87	0.87	36	43	107	40	0.37
20. Whitehead i	126,987	178	357	0.62	0.61	47	33	265	25	0.10
21. Whitehead ii	139,103	194	359	0.19	0.19	32	26	272	14	0.05
22. Whitehead iii	125,918	210	300	0.38	0.41	61	30	290	19	0.07
Other										
23. Rouder i	11,346	52	109	0.37	0.35	50	28	165	19	0.11
24. Rouder ii	16,859	58	145	0.62	0.62	142	72	351	53	0.15
Mean	65,312	145	223	0.52	0.51	59	37	220	27	0.14
Median	46,172	178	197	0.57	0.61	60	31	193	23	0.11

*Note.* All sample sizes and estimates reflect cleaned data. See the Appendix for our cleaning steps which differ from those of the original authors

The fourth and fifth columns provide estimates of reliability. The column labeled “Full” is the sample reliability using all the observations in one group (see Footnote 1); the column labeled “Split” is the split-half reliability. Here, even and odd trials comprised two groups and the correlation of individuals’ effects across these groups was upweighted by the Spearman-Brown prophecy formula. Note that the former estimate is more accurate than the split-half estimate because the former uses variability information across trials, much like in ANOVA, where the later does not.

The next pair of columns, labeled “Sample,” shows the mean sample effect and the standard deviation of individuals’ sample effects around this mean (labled $s_d$). These are sample statistics calculated in the usual way and do not reflect the model. The next two columns are standard deviation estimates from the hierarchical model. The column $\hat{\sigma }$ is the posterior mean for residual variability ($\sigma$ in the model) and the column $\hat{\sigma }_\theta$ is the posterior mean for the true variability across individuals ($\sigma _\theta$ in the model). The final column, labeled $\hat{\gamma }$, is the ratio of these standard deviations. As discussed subsequently, this ratio reflects how reliable the task is and how much the naive correlations will be attenuated.

In hierarchical models, the estimate of true variability across people, $\sigma _\theta$ is smaller than the variability among sample effects ($s_d$ in the table). The reason is straightforward—$s_d$ contains contributions from both individual variability and trial noise. The phenomenon is sometimes called *hierarchical shrinkage* or *hierarchical regularization*, and a brilliant explanation is provided in Efron and Morris (1977). Rouder and Haaf (2019) extend this explanation to inhibition tasks, and the reader is referred to these sources for further discussion.

From the table, we derive the following critical values for the following simulations. We set the trial-by-trial variation to $\sigma = 200$ ms, and the variation of individuals’ true effects to $\sigma _\theta =25$ ms. The critical choice is the latter, and a reader may note its small size especially given the larger value $s_d$, the empirically observed standard deviation of individuals effect scores. The values $s_d$ are larger than $\hat{\sigma }_\theta$ because the former necessarily include contributions from trial noise and variability across individuals. Indeed, the difference $d_i$ is $d_i\sim \text {N}(\mu _\theta ,\sigma ^2_\theta +2\sigma ^2/L),$ with the last term reflecting the contribution of trial noise. Values of $\hat{\sigma _\theta }$ are uncontaminated by trial noise and are the appropriate values for simulating between-participant variability in effects.

Are these studies representative? Representativeness is assessed relative to the goals of the analysis. The goals here are to ascertain representative values of trial variation ($\sigma ^2$) and the true variability in the population after accounting for trial noise ($\sigma ^2_\theta$). We think our 24 studies cover a broad range of values. Contrast, for example, the Hedge et al. flanker study which is characterized by a small degree of trial noise ($\sigma =100$ ms) on one hand, and the Whitehead et al. Stroop studies, which are characterized by a large degree of trial noise ($\sigma \approx 400$ ms) on the other. Likewise, some studies have a low degree of true individual variation while others have a larger degree. Even though there is a broad range of variation, there is much stability in the ratio of true individual variation and trial noise. We think the analysis is novel, highly informative, and forms the new state-of-the art for expectations about trial noise and between-participant variability. Researchers using these tasks need to prepare for an impoverished environment where trial noise is several times larger in standard deviation than true variability across individuals.

## Expected attenuation

The above analysis may also be used to undertstand the degree of attenuation with the usual analysis in Fig. 1. The classical estimate, $\rho ^*$, is given by

$$\begin{aligned} \rho ^* = \rho \left( \frac{L\sigma ^2_\theta }{L\sigma ^2_\theta +2\sigma ^2}\right) , \end{aligned}$$

where *L* is the trial size or number of trials per person per task per condition. This equation is most useful if written with the ratio $\gamma =\sqrt{\sigma _\theta / \sigma }$, with this ratio interpreted as a ratio of signal (true variability) to noise (trial noise). Then, the attenuation factor, $\rho ^*/\rho$ is

1 $$\begin{aligned} \frac{\rho ^*}{\rho } = \left( \frac{L}{L+2/\gamma ^2}\right) . \end{aligned}$$

**Fig. 4:**
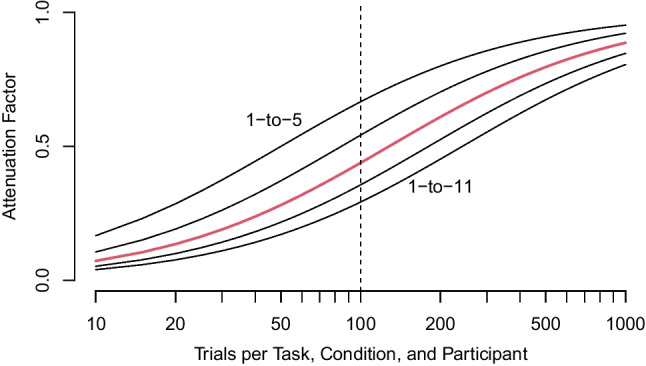
Attenuation of correlation as a function of number of trials (*L*) and signal-to-noise ratio $\gamma$. For typical values ($L=100$, $\eta$=1-to-8), the attenuation is a factor of 44. The plotted values of $\gamma$ are 1-to-5, 1-to-6.5, 1-to-8, 1-to-9.5, and 1-to-11

The last column of Table 2 shows the value of $\gamma$ for the various studies, and the values range from 1/11 to 1/3, with $\gamma =1/8$ corresponding to our typical case. Figure 4 shows the dependence of the attenuation factor on the number of trials (*L*) for various values of signal to noise. As can be seen, with the usual approach of tabulating participant-by-task scores, we expect attenuation to be a factor of .44 for $L=100$ replicates.

## Model-based recovery of correlations among tasks

The critical question is then whether accurate estimation of correlation is possible. The small simulation in the introduction, which was based on the above typical settings for two tasks and a true population correlation of .80, showed that observed correlations among sample effects were greatly attenuated and Spearman’s correction was unstable. We now assess how well observed correlations, Spearman corrections, and hierarchical models localize correlations with larger simulations.

### A hierarchical model for correlation

Here we develop a hierarchical trial-level model for many tasks that explicitly models the covariation in performance among them. A precursor to this model is provided in Matzke et al. (2017) and Rouder and Haaf (2019). A similar mixed-linear model is provides in Whitehead et al. (2020). The difference is that these previous models are applicable for only two tasks and one correlation coefficient. They are not applicable to several tasks and coefficients.

At the top level, the model is:

$$\begin{aligned} Y_{ijk\ell } \sim \text {Normal}(\alpha _{ij}+x_k\theta _{ij},\sigma ^2). \end{aligned}$$

The target of inquiry is $\theta _{ij}$ the effect for the *i*th participant in the *j*th task. The specification is made easier with a bit of vector and matrix notation. Let $\varvec{\theta }_{i}=(\theta _{i1},\ldots ,\theta _{iJ})'$ be a column vector of the *i*th individual’s true effects. This vector comes from a group-level multivariate distribution. The following is the case for three tasks:

$$\begin{aligned} \varvec{\theta }_i=\begin{bmatrix} \theta _{i1}\\ \theta _{i2} \\ \theta _{i3} \end{bmatrix} \sim \text {N}_3 \left( \begin{bmatrix} \mu _1\\ \mu _2\\ \mu _3 \end{bmatrix}, \begin{bmatrix} \sigma ^2_{\theta _1} &{} \rho _{12}\sigma _{\theta _1}\sigma _{\theta _2} &{} \rho _{13}\sigma _{\theta _1}\sigma _{\theta _3}\\ \rho _{12}\sigma _{\theta _1}\sigma _{\theta _2} &{} \sigma _{\theta _2}^2 &{} \rho _{23}\sigma _{\theta _2}\sigma _{\theta _3}\\ \rho _{13}\sigma _{\theta _1}\sigma _{\theta _3} &{} \rho _{23}\sigma _{\theta _2}\sigma _{\theta _3} &{} \sigma _{\theta _3}^2\\ \end{bmatrix} \right) . \end{aligned}$$

More generally, for *J* tasks,

2 $$\begin{aligned} \varvec{\theta }_i \sim \text {N}_J(\varvec{\mu },\varvec{\Sigma }_\theta ). \end{aligned}$$

Priors are needed for $\varvec{\mu }$, the vector of task means, and $\varvec{\Sigma }_\theta$, the covariance across the tasks. We take the same strategy of using scientifically-informed priors. For $\varvec{\mu }$, we place the normal in Fig. 3A on each element. For $\varvec{\Sigma }_\theta$, the classic choice is the *inverse Wishart* prior. This choice is popular because it is flexible and computationally convenient (O’Hagan and Forster, 2004). The inverse Wishart requires a scale parameter, and we set it so that the marginal prior on standard deviations of true variation matches the distribution in Fig. 3B.^2^ It is the use of the inverse Wishart here that allows the model to be applicable to many tasks and correlation coefficients.

**Fig. 5:**
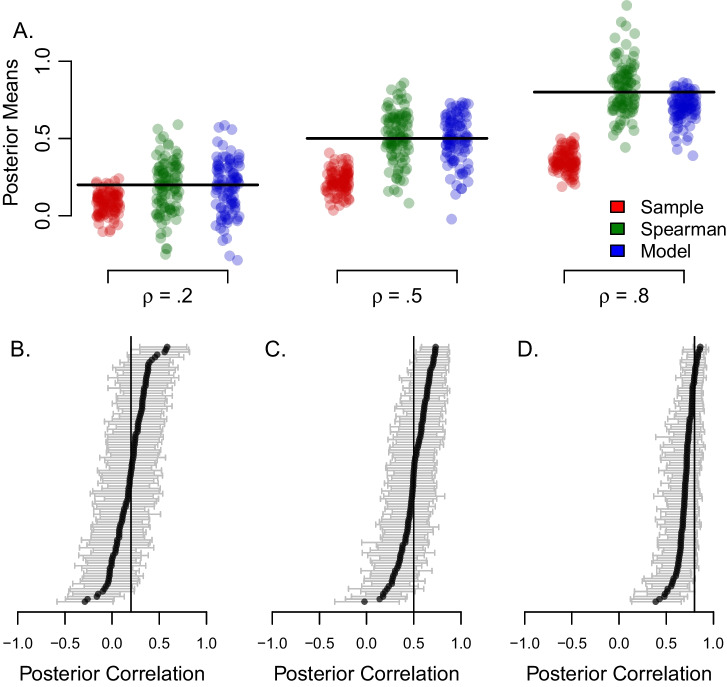
Revocery of correlations from two tasks. A: Boxplots of recovered correlations from sample correlations, Spearman’s correction, and the hierarchical model. B-D: Posterior 95% credible intervals for the model-recovered correlations for true correlations of .2, .5, and .8, respectively

### Two tasks

Can correlations be localized in typical tasks?

The first simulation is for two tasks. In performing simulations, we must set the sample sizes, ground truth relations among the tasks, trial noise and true individual variation. The former two were set by the preceding analysis. For all of our simulations, we used $I=200$ people and $L=100$ replicates per condition. We think these are good choices to emulate designs where many individuals are going to run in several inhibition tasks. For tasks with two conditions, there are 40,000 observations per task. In a typical battery with $J=10$ tasks, the total number of observations is 400,000, which is quite large. Hence, our choices seem appropriate to typical large-scale individual-difference studies with experimental tasks.

Using the typical sample sizes discussed above, each hypothetical data set consisted of 80,000 observations (200 people $\times$ 2 tasks $\times$ 2 conditions $\times$ 100 replicates per condition). The last input to the simulations is the true correlation across the two tasks. This value were varied through levels of .2, .5, and .8. For each of these levels, 100 data sets were simulated and analyzed. Figure 5A shows the results. The correlations from participant-by-task sample means are shown in red, and are called here “sample correlations.” As expected, these correlations suffer a large degree of attenuation from trial noise. Correlation estimates from Spearman’s correction are shown in green. These values are better centered though some of the corrected values are greater than 1.0. The correlation estimates from the hierarchical model are shown in blue.

Overall, the correlation estimates from Spearman’s correction and the hierarchical model have less bias than sample correlations. Yet, the estimates are quite variable. For example, consider correlations when the population value is .2. The model estimates range from -0.29 to 0.58 and miss the target with a RMSE of 0.17. Spearman corrected estimates are a slightly better and have an RMSE for this case of 0.17. Overall though, this variability is quite high especially given the large number of observations. The correlations are not well localizedm and we would not have confidence in substantive conclusions with it.

**Fig. 6:**
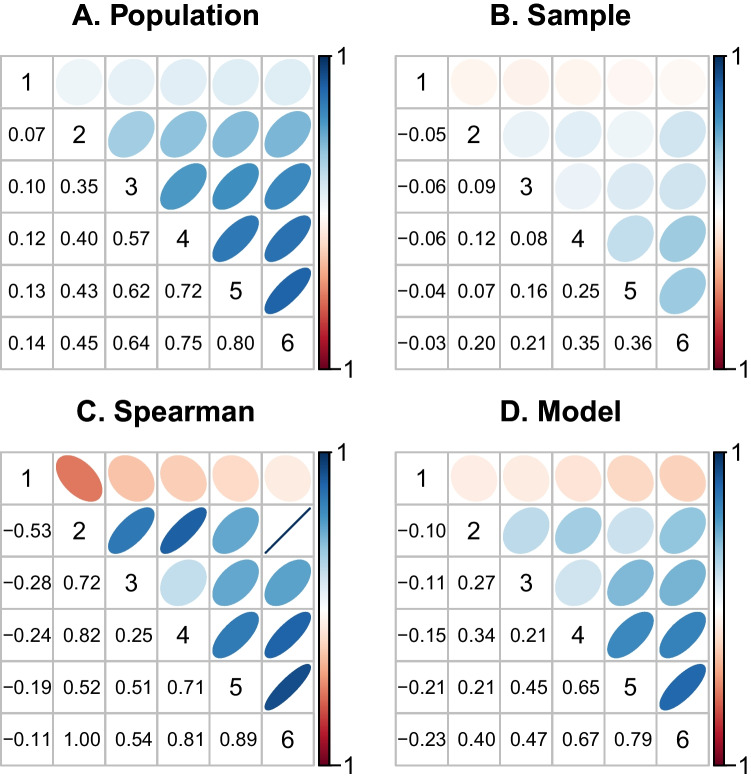
True and recovered correlation matrices for six tasks. A: True population correlations. B-D: Correlation estimates from a single run

Figure 5A shows only posterior mean estimates. Yet, in the Bayesian approach, the target is not just the posterior mean, but the entirety of the posterior distribution. Figure 5B-D shows the posterior 95% credible intervals for all runs with true correlations of .2, .5, and .8, respectively. There are two noteworthy trends. First, the 95% credible intervals tend to contain the true value on 89% of the simulation runs. This means that the posterior variability is relatively well calibrated and provides reasonably accurate information on the uncertainty in the correlation. Second, there is a fair amount of uncertainty meaning that the analyst knows that correlations have not been well localized. With the Bayesian model-based estimates, at least we know how uncertain we are in localizing true correlations. With sample correlation and with the Spearman correction, we have no such knowledge.

### Six tasks

We explored correlations across six tasks. Each hypothetical data set consisted of 240,000 observations. To generate a wide range of correlations, we used a one-factor model to simulate individuals’ true scores. This factor represents the individual’s inhibition ability. This ability, denoted $z_i$, is distributed as a standard normal. Tasks may require more or less of the individuals’ inhibition ability. Therefore, task loadings onto this factor $z_i$ are variable and, as a result, a wide range of correlations occur. The following task loading values work well in producing a diversity of correlations: 1.5 ms, 5.7 ms, 9.9 ms, 14.1 ms, 18.3 ms, and 22.5 ms. Following the one-factor structure we may generate true scores, $\theta _{ij}$, for each task and participant:

$$\begin{aligned} \theta _{ij} \sim \text {Normal}(\mu _j+z_iw_j,\eta ^2), \end{aligned}$$

where $z_i$ is the true ability, $w_j$ is the task loading, $\mu _j$ is the task overall mean, and $\eta ^2$ is residual variability in addition to that from the factors. In simulation we set $\eta =10$ ms, and this setting yields standard deviations across $\theta _{ij}$ between 10 ms and 30 ms, which is similar to the 25 ms value used previously. The true population variance for the one-factor model is $\varvec{\Sigma } = \varvec{w}\varvec{w'} + \varvec{I}\eta ^2$, where $\varvec{w}\varvec{w'}$ is the matrix formed by the outer product of the task loadings. The true correlation matrix from the variance-covariance matrix $\varvec{\Sigma }$ is shown in Fig. 6A, and the values subtend a large range from near zero to 0.80.

**Fig. 7:**
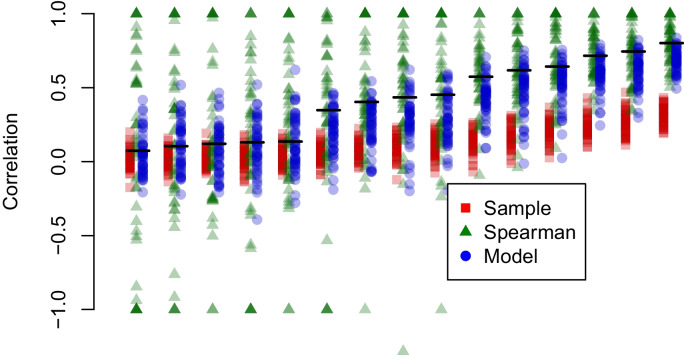
Recovery of correlations from six tasks. True correlations are derived from a one-factor model and are displayed in Figure 6

**Fig. 8:**
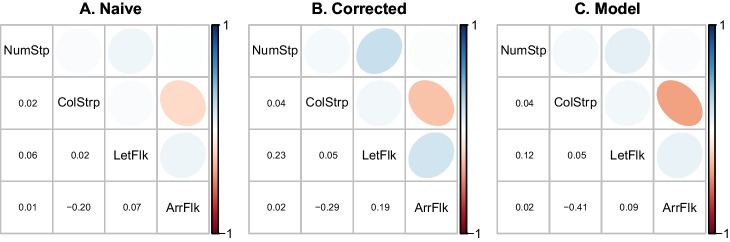
Correlations among select tasks in the Rey-Mermet data set. Tasks are a number Stroop task, a color Stroop task, a letter flanker task, and an arrow flanker task. Details of the tasks are provided in the Appendix

The recovery of correlations is shown for a single simulation run in Fig. 6B-D. The attenuation for the sample correlations is evident, as is variability in model-based and Spearman corrected estimates. Figure 7 shows the performance of the methods across the 50 simulation runs. As can be seen, there remains the dramatic attenuation for the sample correlation of sample effects and excessive variability for the Spearman-corrected and model-based correlation estimates. Spearman corrected estimates are free to be outside the valid range from -1 to 1. We imagine that any researcher encountering these values could justifiably set them to the appropriate endpoint, and we do so in Fig. 7. Nonetheless, the RMS errors remain high—across the whole range of true values they are 0.59 and 0.19 for the Spearman correlation and model, respectively. It is somewhat heartening that model recovery is somewhat informative.

**Fig. 9:**
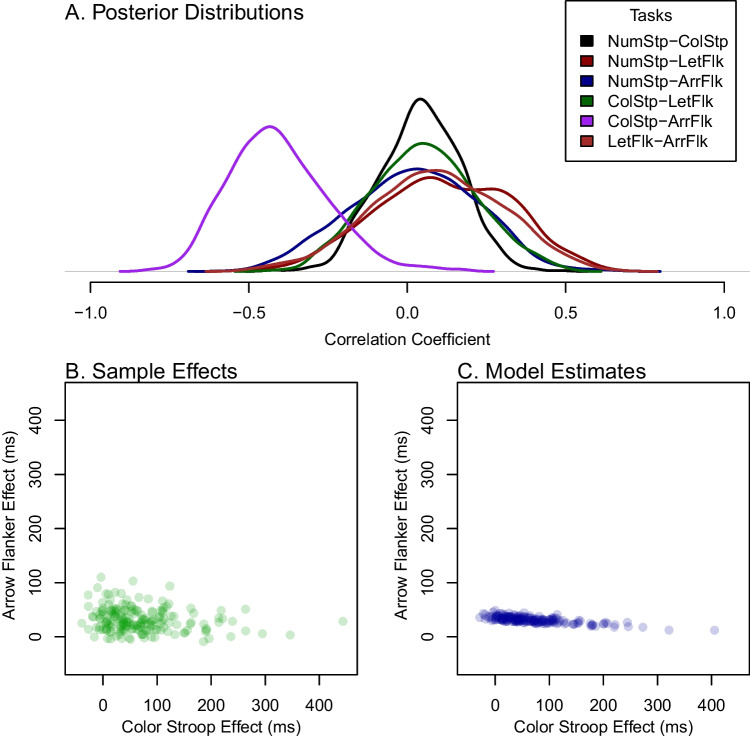
A. Model-based posterior distributions of population correlations among tasks. The large variance shows the difficulty of recovery. B. Individuals’ sample effects for color Stroop and arrow flanker tasks show. C. Hierarchical model estimates show a large degree of shrinakge for arrow flankers but not for color Stroop reflecting the increased range of color Stroop effects

## Analysis of Rey-Mermet, Gade, and Oberauer, (2018)

To assess real-world correlation recovery, we re-examined the flanker and Stroop tasks in Rey-Mermet et al.’s battery of inhibition tasks. The authors included two different types of Stroop tasks (a number Stroop and a color Stroop task, see the Appendix for details) and two different types of flanker tasks (a letter flanker and an arrow flanker task, see the Appendix for details). The question then is about the correlation across the tasks.^3^

The top three rows of Fig. 8 show the estimated correlations from sample effects, Spearman’s correction, and the hierarchical model. Given the previous simulations results, it is hard to know how much credence to give these estimated correlations. In particular, it is hard to know how to interpret the negative correlation between the arrow flanker and color Stroop task.

To better understand what may be concluded about the range of correlations, we plot the posterior distribution of the correlation (Fig. 9A). These distributions are unsettling. The variation in most of these posteriors is so wide that firm conclusions are not possible. The exception is the null correlation between number and color Stroop which seems to be somewhat well localized. The surprisingly negative correlation between color Stroop and arrow flanker comes from a posterior so broad that the 95% credible interval is [-0.27,0.39]. Here, all we can say is that very extreme correlations are not feasible. We suspect this limited result is not news.

Analysis of Rey-Mermet et al. (2018) provides an opportunity to examine how hierarchical models account for variation across trials as well as variation across people. Figure 9B shows sample effects across individuals for the color Stroop and arrow flanker tasks, the two tasks that were most negatively correlated. There is a far greater degree of variation in individual’s effects for the color Stroop task than for the arrow flanker task. The model estimates (Fig. 9C) reflect this difference in variation. The variation in arrow flanker is so small that it can be accounted for with trial variation alone. As a result, the hierarchical model shows almost no individual variability. In contrast, the variability in the color Stroop is large and the main contributor is true variation across individuals rather than trial variation. Hence, there is relatively little shrinkage in model estimates. The lack of variation in the arrow flanker task gives rise to the uncertainty in the recovered correlation between the two tasks.

## General Discussion

One basic question facing researchers in cognitive control is whether inhibition is a unified phenomenon or a disparate set of phenomena. A natural way of addressing this question is to study the pattern of individual differences across several inhibition tasks. In this paper, we have explored whether correlations across inhibition tasks may be localized. We consider typically large studies that enroll hundreds of participants and run tasks with 100s of usable trials per condition. Our main assessment is downbeat—correlations across typical inhibition tasks, say Stroop, flanker, Simon, and the like, are difficult to localize. This statement of poor localization holds for hierarchical models that model trial noise.

Why this depressing state-of-affairs occurs is fairly straightforward. Relative to trial noise, there is little true individual variation in inhibition tasks. To see why this is so, consider an average effect, say one that is 60 ms. In inhibition tasks like Stroop and flanker, we can safely make a *dominance assumption*—nobody truly has a negative effect (Haaf and Rouder, 2017). That is to say nobody truly identifies incongruent stimuli faster than congruent ones. Under this assumption, where all true scores are positive, a small mean necessarily implies a small variance. For example, if true Stroop effects are reasonably normally shaped, the mean is 60 ms and there can be no mass below zero, then an upper bound on variability across true scores is a standard deviation of 25 ms or so. This is a small amount of variation compared to trial variability, which is typically 8 times larger. This small degree of true variation necessarily implies a small degree of covariation across tasks. And this small degree of covariation is beyond the resolution of typical experimental designs with limited numbers of trials.

We believe this problem of localizing individual differences and correlations extends beyond inhibition tasks. It likely holds broadly in most task domains as most tasks have relatively small effects, whether on the order of 60 ms for RT, on the order of .08 for accuracy, or maybe on the order of 1/10th of the scale for Likert values. If we make a dominance assumption—each individual has a true effect in the same direction—then there cannot be much individual variability else these mean effects would be larger. And measuring correlations with small degrees of individual variability may be beyond the resolution of typical designs.

### Recommendations

Based on the above correlation-localization results, we can make the following recommendations:

*Be mindful of attenuation*. Researchers have certainly been aware of measurement error and understand the link between measurement error and attenuation. Yet, they nonetheless estimate correlations in high trial-noise environments? Previous to this work, there were no systematic studies of the degree of attenuation in inhibition, and hence little basis to understand its effects. Here, we argue that the critical factor—the ratio of true variability to trial noise—is on the order of 1-to-8, and may be as great as 1-to-11. Now, for various numbers of trials, researchers can compute how much attenuation is expected using Equation (1). These values can be used for sample size planning and as context in interpretation.

*Stress the number of trials in a task*. Typically, researchers are quick to report the number of participants they have run. These numbers appear not only in method sections, but in abstracts and tables. And researchers may believe that with larger numbers of participants, results are better powered and become more accurate. This belief is wrong, especially in high trial noise environments. The more critical design element is the number of trials per person within a task. With few trials, there is much trial noise and much attenuation. Low numbers of trials add systematic bias whereas low numbers of people add unsystematic noise. Moreover, using high numbers of participants with low numbers of trials breeds high confidence in a wrong answer. We recommend researchers consider running fewer tasks and conditions to gain larger numbers of trials per task. Moreover, we recommend researchers stress the role of the number of trials in their discussion and report these numbers in their method sections, tables, and abstracts.

*Localization is much better in measures.* We have focused here on experimental tasks where there is a theoretically-motivated contrast between conditions. The contrast is key—it allows isolation of the process of interest, say cognitive control, from other factors such as motivation or general speed. The claim here is that correlations among tasks are difficult to localize

The alternative is to use a measure rather than a task. Measures have better statistical properties than tasks. They are often highly reliable and lead to higher correlations among similarly-motivated measures (Draheim et al. 2019; Draheim, Tsukahara, Martin, Mashburn, and Engle, 2021). It is far easier to localize correlations with measures than tasks.

For us, however, interpretability remains an issue. Whether a certain measure indexes a given process is asserted *prima facie* rather than by experimental logic. Some assertions seem quite reasonable, say that performance on a span task indexes working memory (Daneman and Carpenter, 1980). Others seem less reasonable. We worry, for example, that antisaccade accuracy (Kane et al., 2001) reflects the speed of detecting briefly flashed targets (general speed) as much as suppressing a cue located away from the target (cognitive control).

In practice, it is sometimes difficult to keep track of what is a task and what is a measure if only because we tend to use “task” for both tasks and measures. Popular measures such as the antisaccade accuracy measure and the stop-signal measure are routinely called tasks, but, in our usage, they are not. Regardless of terminology, researchers need be aware of a foundational trade-off: tasks have high interpretability and poor statistical properties to index individual differences while measures have negotiated interpretability and good statistical properties.

### Strategies for better correlation recovery

The above recommendations center on understanding how much variability and bias there is in recovering latent correlations. But they do not address the difficult situation head on. How can we improve the recovery? We consider the following possibilities:

*More Trials.* A seemingly simple solution is to run more trials per person per condition. The usual 50 or 100 trials per task per condition is clearly not enough. Here is a seat-of-the-pants calculation to show what might be ideal: Suppose we wish to localize individual effects up to a maximum standard error of 10 ms. With this value, we can calculate the number of needed trials. If people have 200 ms of trial-level noise, and we are computing a difference score, then the standard error is $200\sqrt{2/L}$, where *L* is the number of trials per condition per task. Setting this standard error to 10 ms yields $L=800$, or about 1,600 trials per task per participant.

Now such a large number will assuredly prove problematic for several reasons. Participants tire and lose motivation. The target effects themselves may attenuate with excessive numbers of trials (Davidson, Zacks and Williams, 2003; Dulaney and Rogers 1994). Researchers may not have resources to run large number of trials per individual per task, and even if the resources are available, such designs may not be practical. Still, at least from a statistical point-of-view, more trials is always better than less so long as those trials result in comparable behavior across the sessions. Researchers using more trials do need to check for fatigue, loss of effect, loss of motivation and the like.

As an aside, we recommend researchers never run neutral conditions. The contrast between incongruent and congruent is far more important, and performance on neutral trials do not enter into correlational structures. Removing neutral conditions allows for larger numbers of congruent and incongruent trials. If researchers wish to critically assess whether the neutral condition is more like the incongruent or the congruent condition, they should do so outside an individual-differences design.

*Better Tasks Through Gamification.* Perhaps the most obvious solution is to search for inhibition tasks with greater individual variation. In practice, this means engineering tasks to have large overall effects with relatively small trial noise. One innovation in cognitive control is the use of so-called *gamified* tasks (Deveau, Jaeggi, Zordan, Phung and Seitz, 2015; Kucina et al. 2022; Wells et al. 2021). When a task is gamified, it is made into a video game. There may be sound, color, theme music, point scores, leaderboards, and other elements of video-game play. There are two possible advantages of gamification. The first is that gamified tasks may be more reliable in that they have higher signal-to-noise ratios, $\gamma$ (Kucina et al., 2022; Wells et al., 2021). For example, Wells et al. (2021) claim that the increased arousal and engagement from gamification results in more reliable data. Kucina et al. (2022) note that it may be possible to have combined stimulus elements in gamified settings that increase conflict effects. The second possible advantage is that people may be willing to engage with a gamified task at a higher level for longer (Deveau et al., 2015). Gamification then may be an effective tactic for increasing trial size without tears.

*Combined Dependent Measures*. Another approach is to refine how we use dependent variables. A new trend is to consider both speed and accuracy in combination through a diffusion model (Enkavi et al. 2019; Hedge, Powell, Bompas, and Sumner, 2021; Weigard, Clark and Sripada, citeyearrefspsWeigard.etal.2021). There are two possible advantages: first, by considering speed and accuracy jointly, individual differences in the speed-accuracy tradeoff may be considered and modeled. Second, resulting parameters such as the rate of evidence accumulation may be more sensitive and less affected by trial noise than RT or accuracy alone (Lerche et al., 2020; Weigard et al., 2021). This claim, however, is controversial as Enkavi et al. (2019) found only marginally higher reliability coefficients for drift rates vs. response time alone.

### Modeling trial noise

Is it worth it to use hierarchical models to account for trial noise? Based on this report, the answer may be “not yet.” These models lead to only marginally better localization of correlations. Currently, the main advantage is that one can assess the degree of localization. Hierarchical models provide a useful measure of uncertainty.

The main problem with the hierarchical models presented here is that they stop at covariance. They do not lend themselves to latent-variable decomposition of covariance such as that in factor models. Researchers who adopt these trial-noise models seemingly give up the power of latent-variable modeling. It’s not a good trade.

One future direction is the development of trial-level confirmatory latent-variable models. For example, if we are interested in the basic question whether there is a unified concept of inhibition, we might develop a trial-level one-factor model or a trial-level bifactor model. The good news here is that these models offer constraint over the Wishart priors used here. With this constraint, it may be possible to better localize correlations among tasks. Second, and perhaps more importantly, localization of correlations may become secondary to model assessment and model comparison. How well does one trial-level confirmatory structure compare to another?

We are not that far from trial-level confirmatory factor models. Key to this endeavor is the work of Merkel and colleagues (Merkle, Fitzsimmons, Uanhoro and Goodrich, 2021; Merkle and Rosseel, 2018) who have been studying the most efficient approaches to Bayesian structural-equation modeling. Their package blavaan uses lavaan syntax, which is well known and quite convenient. It seems that extensions to trial noise are possible though not yet developed.

### Concluding thought

We show here that it is difficult to address whether inhibition is a unified or disparate concept using individual differences with experimental tasks. Simply put, we cannot as of yet tell if the low correlations with conventional aggregation reflect attenuation from excessive trial noise or a true lack of covariation. Without the benefit of better methods and experiments, we offer no critique of or advocacy for extant theories of cognitive control.

Solving the difficulties with tasks is going to entail larger experiments, perhaps better tasks, and perhas not-yet-developed trial-level latent-variable confirmatory models. We hope this paper lays a foundation for understanding what is at stake and motivates the needed developments. Although the message is disheartening in the short run, we think there is reason to be optimistic in the long run. Given the talent in the field, individual-difference researchers are going to rise to the challenge because these solutions may well be within our grasp.

## Appendix

### Data Set 1, Von Bastian, Souza, and Gade (2015)

The task was a number Stroop task. Participants were presented a string of digits. In each string, the digits were always replicates, say *22* or *444*, and the lengths varied from one digit to four digits. The participants identified the length of the string, for example, the correct report for *444* is 3. In the congruent condition, the length and the digits matched; e.g., *22* and *4444*. In the incongruent condition, the length and digits mismatched, e.g., *44* and *2222*. We used somewhat different data cleaning steps than the original authors. Ours are described in Haaf and Rouder (2017).

### Data Set 2, Hedge et al. (2018)

The task was a color Stroop task. Participants identified the color of a centrally presented word (red, blue, green, or yellow). In the congruent condition, presentation color and word meaning matched. In the incongruent condition, they did not match. Following Hedge et al. (2018), we combined data from their Experiments 1 and 2. Our cleaning steps differed from Hedge et al. (2018) and are described in the code accompanying (Rouder and Haaf, 2019). Briefly, we discarded participants who had an error rate greater than 10%.

### Data Set 3, Pratte, Rouder, Morey, and Feng (2010), Experiment 1

The task was a color Stroop task. Participants identified the color of the color words, e.g. the word *RED* presented in blue. In the congruent condition, presentation color and word meaning matched, e.g. *BLUE* presented in blue. In the incongruent condition, they did not match, e.g. *RED* presented in blue. Cleaning steps were those from the original authors as implemented in their analysis code.

### Data Set 4, Pratte et al. (2010), Experiment 2

The task was a sidedness judgment Stroop task. Participants were presented the words *LEFT* and *RIGHT*, and these were presented to the left or right of fixation. Participants identified the position of the word while ignoring the meaning of the word. A congruent trial occurred when position of the word and word meaning corresponded; an incongruent trial emerged when position and word meaning did not correspond. Cleaning steps were those from the original authors as implemented in their analysis code.

### Data Set 5, Rey-Mermet et al. (2018)

The task was a number Stroop task. Participants identified the length of digit strings much like in Data Set 1. Cleaning proceeded as follows. First, note that in the original, trials ended at 2.0 seconds even if the participant did not respond. We call these trials *too slow*. 1. We discarded the five participants discarded by the original authors; 2. We discarded too-slow trials, error trials, and trials with RTs below .275 seconds (*too-fast* trials). 3. We discarded all participants who had more than 10% errors, who had more than 2% too-slow trials, or more than 1% too fast trials.

### Data Set 6, Rey-Mermet et al. (2018)

The task was a color Stroop task. Participants identified the color of the presented words (red, blue, green, or yellow). The presentation color and word meaning matched in the congruent condition and did not match in the incongruent condition. Cleaning steps were those from the original authors as implemented in their analysis code.

### Data Set 7, Whitehead, Brewer, and Blais (2019) (Experiment 1)

The task was a color Stroop task similar to Data Set 6. Cleaning steps were those from the original authors as implemented in their analysis code.

### Data Set 8, Whitehead et al. (2019) (Experiment 2)

The task was a color Stroop task similar to Data Set 6. The ratio of congruent-to-incongruent items was 3-to-1 rather than 1-to-1. Cleaning steps were those from the original authors as implemented in their analysis code.

### Data Set 9, Whitehead et al. (2019) (Experiment 3)

The task was a color Stroop task similar to Data Set 6. The word-to-color contingencies were manipulated to remove certain feature overlaps. Cleaning steps were those from the original authors as implemented in their analysis code.

### Data Set 10, Von Bastian et al. (2015)

The task was a Simon task. Participants were presented either a green or red circle to the left or right of fixation. They identified the color, green or red color by pressing buttons with their left or right hand, respectively. The spatial location of the circle and of the response could be either congruent (e.g., a green circle appearing on the left) or incongruent (e.g., a green circle appearing on the right). Cleaning steps are described in Haaf and Rouder (2017).

### Data Set 11, Pratte et al. (2010), Experiment 1

The task was a Simon task almost identical to that in Data Set 10. Participants identified the color of a square presented to the left or right of fixation by making a lateralized key response. A congruent trial occurred when position of the square was ipsilateral correct key response.; an incongruent trial occurred when the position of the square was contralateral to the correct key response. Cleaning steps were those from the original authors as implemented in their analysis code.

### Data Set 12: Pratte et al. (2010), Experiment 2

The task was a *lateral-words* Simon task. Participants were presented the words *LEFT* and *RIGHT* to the left or right of fixation. Participants identified the meaning of the word while ignoring the location of the word. A congruent trial occurred when position of the word and word meaning corresponded; an incongruent trial occurred when position of the word and word meaning did not match. Cleaning steps were those from the original authors as implemented in their analysis code.

### Data Set 13, Whitehead et al. (2019) (Experiment 1)

The task was a location Simon task similar to Pratte et al. (2010). Directional words UP, DOWN, LEFT, RIGHT were placed at locations. Participants ignored the location and reported the meaning of the word. Cleaning steps were those from the original authors as implemented in their analysis code.

### Data Set 14, Whitehead et al. (2019) (Experiment 2)

The task was a location Simon task similar to Data Set 13. The ratio of congruent-to-incongruent items was 3-to-1 rather than 1-to-1. Cleaning steps were those from the original authors as implemented in their analysis code.

### Data Set 15, Whitehead et al. (2019) (Experiment 3)

The task was a color Stroop task similar to Data Set 6. The word-to-location contingencies were manipulated to remove certain feature overlaps. Cleaning steps were those from the original authors as implemented in their analysis code.

### Data Set 16, Von Bastian et al. (2015)

The task was a letter-flanker task. Participants were presented strings of seven letters and judged whether the center letter was a vowel (*A*, *E*) or consonant (*S*, *T*). The congruent condition was when the surrounding letters came from the same category as the target (e.g. *AAAEAAA*); the incongruent condition was when the surrounding letters came from the opposite category of the target (e.g., *TTTETTT*). Cleaning steps are described in Haaf and Rouder (2017).

### Data Set 17: Hedge et al. (2018)

The task was an arrow flanker task almost identical to Data Set 11. Following Hedge et al. (2018), we combined data from their Experiments 1 and 2. Our cleaning steps differed from Hedge et al. (2018) and are described in Rouder and Haaf (2019).

### Data Set 18, Rey-Mermet et al. (2018)

The task was an arrow flanker task. Participants identified the direction of the central arrow (left/right) while ignoring four flanking arrows. Congruency and incongruency occurred when the center arrow matched and mismatched the direction of the flanker arrows, respectively. Cleaning steps were the same for Data Set 5.

### Data Set 19, Rey-Mermet et al. (2018)

The task was a letter-flanker task almost identical to Data Set 18. Cleaning steps were the same for Data Set 5.

### Data Set 20, Whitehead et al. (2019) (Experiment 1)

The task was a letter flanker task where participants identified a central letter. Cleaning steps were those from the original authors as implemented in their analysis code.

### Data Set 21, Whitehead et al. (2019) (Experiment 2)

The task was similar to Data Set 20. The ratio of congruent-to-incongruent items was 3-to-1 rather than 1-to-1. Cleaning steps were those from the original authors as implemented in their analysis code.

### Data Set 22, Whitehead et al. (2019) (Experiment 3)

The task was a letter flanker task similar to Data Set 20. Target-to-distractor letter contingencies were manipulated to remove certain feature overlaps. Cleaning steps were those from the original authors as implemented in their analysis code.

### Data Set 23, Rouder, Lu, Speckman, Sun, and Jiang (2005)

The task was a digit-distance task. Participants were presented digits 2, 3, 4, 6, 7, 8, and had judged whether the presented digit was less-than or greater-than five. Digits further from five are identified faster than those close to 5. Responses to digits 2 and 8 comprised the *far* condition; responses to digits 4 and 6 comprised the *close* condition. The difference in conditions comprised a *distance-from-five* effect. Cleaning steps were those from the original authors as implemented in their analysis code.

### Data Set 24, Rouder, Yue, Speckman, Pratte, and Province (2010)

The task was a grating-orientation discrimination. Participants were presented nearly-vertical Gabor patches that were very slightly displaced to the left or right; they indicated whether the displacement was left or right. Displacements were $\pm 1.5^\circ$, $\pm 2.0^\circ$, and $\pm 4.0^\circ$ from vertical. Responses from the $\pm 1.5^\circ$ comprised the *hard* condition; responses from the $\pm 4.0^\circ$ comprised the *easy* condition; the difference comprised a *orientation-strength* effect. Cleaning steps were those from the original authors as implemented in their analysis code.

## Footnotes

1 The estimation of reliability in tasks is different than the estimation of reliability in a classical test because there are replicates within people and conditions in tasks. The presence of these replicates may be leveraged to produce better estimates of error variability than when they are not present. Let $\bar{Y}_{ik}$ and $s_{\bar{y}_{ik}}$ be the sample mean and sample standard error for the *i*th individual in the *k*th condition, $k=1,2$. Let $d_{i}=\bar{Y}_{i2}-\bar{Y}_{i1}$ be the effect for the *i*th individual, and let $V_d$ be the sample variance of these effects. This sample variable is the total variance to be decomposed into true and error variances. Assuming an equal number of trials per condition, the error variance for the *i*th person, denoted $V_{ei}$ is $s^2_{\bar{y}_{i1}}+s^2_{\bar{y}_{i2}}$. The estimate of error variance is simply the average of these individual error variances, or $V_e={\sum }_i{\sum }_k s_{\bar{y}_{ik}}^2/I$. The reliability is $r=(V_d-V_e)/V_d$.

2 There is an alternative choice of prior for covariance that we extensively explored, the *LKJ prior* (Lewandowski, Kurowicka, and Joe, 2009). This prior is less informative than the Wishart because, unlike the Wishart, the estimation of correlation is independent of the specification of scale. Consequently, this prior is recommended (McElreath, 2016), and implementation is convenient in the R-package rstan (Stan Development Team, 2018). Yet, we found better performance for the inverse Wishart in simulations in that the posterior credible intervals were smaller and better covered the true value. The increased performance of the Wishart reflects the fact that researchers have a rough idea about the scale of individual differences—it is on the order of tens of milliseconds—and this is enough information for the improved performance of the inverse Wishart.

3 One of the elements that makes analysis complicated is how to exclude low-performing participants. In the previous analysis, where each task was analyzed in isolation, we retained all participants in a task who performed over 90% accuracy on that task. In the current analysis, however, we must have the same participants for all four tasks. We decided to retain those participants who have over 90% accuracy on all four tasks. With this strict criterion, we retain only 180 of the original 289 participants. The most noticeable effect of this exclusion is that the reliability for the arrow flanker task was reduced from .87 to .56. The fact that the reliability changes so much indicates that the high reliability was driven by a few participants with very large difference scores. This cutoff differs from Rey-Mermet et al. (2018), who used a 75% accuracy. With this lower cutoff, they included many more participants.
